# Glucagon-like peptides 1 and 2 and vasoactive intestinal peptide are neuroprotective on cultured and mast cell co-cultured rat myenteric neurons

**DOI:** 10.1186/1471-230X-12-30

**Published:** 2012-04-01

**Authors:** Ulrikke Voss, Elin Sand, Per M Hellström, Eva Ekblad

**Affiliations:** 1 Department of Experimental Medical Science, BMC B11, Lund University, SE-22184 Lund, Sweden; 2 Department of Medical Sciences, Uppsala University, SE-75185 Uppsala, Sweden

## Abstract

**Background:**

Neuropathy is believed to be a common feature of functional and inflammatory intestinal diseases. Vasoactive intestinal peptide (VIP) is an acknowledged neuroprotective agent in peripheral, including enteric, and central neurons. The proglucagon-like hormones glucagon-like peptide 1 and 2 (GLP1 and GLP2) belong to the secretin/glucagon/VIP superfamily of peptides and GLP1 and GLP2 receptors are expressed in enteric neurons. Possible neuroprotective effects of these peptides were investigated in the present study.

**Methods:**

GLP1, GLP2 and VIP were added to cultured myenteric neurons from rat small intestine or to co-cultures of myenteric neurons and rat peritoneal mast cells. Receptor selectivity was tested by the simultaneous presence of a GLP1 receptor antagonist (exendin (9-39) amide) or a VIP receptor antagonist (hybrid of neurotensin 6-11 and VIP 7-28). Neuronal survival was examined using immunocytochemistry and cell counting.

**Results:**

GLP1, GLP2 and VIP significantly and concentration-dependently enhanced neuronal survival. In addition the peptides efficiently counteracted mast cell-induced neuronal cell death in a concentration-dependent manner. Exendin(9-39)amide reversed GLP1-induced neuroprotection while GLP2- and VIP-induced enhanced neuronal survival were unaffected. The VIP receptor antagonist reversed GLP1- and VIP-induced neuroprotection while the GLP2-induced effect on neuronal survival was unaffected.

**Conclusions:**

By activating separate receptors VIP, GLP1 and GLP2 elicit neuroprotective effects on rat myenteric neurons cultured with or without mast cells. This implies a powerful therapeutic potential of these peptides in enteric neuropathies with a broad spectrum of applications from autoimmunity to functional disorders.

## Background

The enteric nervous system (ENS) is pivotal in the regulation and coordination of gastrointestinal (GI) motility, secretion and blood flow. GI discomfort such as nausea, bloating, abdominal pain, constipation, diarrhea or delayed gastric emptying are common features in functional bowel diseases affecting, in particular, a large number of diabetic patients [1]. The pathogenesis of GI symptoms in diabetes is not fully understood but autonomic neuropathy and abnormal glucose levels are suggested to be important. In diabetic rats neurodegeneration [2] and apoptosis [3] occur in myenteric neurons. Oxidative stress is considered the most important factor in causing diabetes-induced enteric neuropathy [4,5]. Collectively these studies indicate the occurrence of diabetes-related neurodegenerative processes in the ENS that eventually lead to neuropathy, intestinal dysfunction and GI discomfort.

Glucagon-like peptide 1 (GLP1) attracts much attention due to its effects on glucose-stimulated insulin secretion, beta-cell proliferation and food intake. It is currently in clinical use in patients with type II diabetes in order to treat hyperglycemia and a number of beneficial side effects e.g. weight loss due to better satiety control [for a recent review see [6]] and protection of beta-cells against cytokine-mediated apoptosis [7] have been recognized. A number of observations also indicate that GLP1 is neuroprotective in the central nervous system [8] and clinical trials on patients with Parkinsons disease are announced [9]. In the peripheral nervous system GLP1 prevents experimentally induced sensory neuropathy [10,11]. In this context it is of interest to note that GLP1 given to patients with irritable bowel syndrome provides an effective, on demand, relief of acute pain attacks [12].

The incretin GLP1 is encoded within the proglucagon precursor and released, together with glucagon-like peptide 2 (GLP2) in a 1:1 ratio, from L-cells in the ileum and colon in response to food ingestion. GLP2 acts as an intestinotrophic factor mainly by stimulating crypt cell proliferation but it also possesses anti-apoptotic effects and enhances nutrient absorption. Protective effects of GLP2 have been explored in clinical entities like short bowel syndrome, total parenteral nutrition-induced intestinal atrophy and in inflammatory bowel disease (IBD) [for a review see [13]]. In trinitrobenzene sulfonic acid or dextran sodium sulfate models of IBD GLP2 treatment reduces the intestinal inflammation and counteracts inflammation-induced loss of enteric neurons. In addition, GLP2 treatment increases the number of vasoactive intestinal peptide (VIP)-expressing enteric neurons and the possibility that GLP2 effects are mediated via release of VIP is suggested [14,15]. VIP exhibits established neuroprotective properties in peripheral, including enteric, and central neurons [16-20].

Aims of the present study were to investigate possible neuroprotective effects of GLP1, GLP2 and VIP on myenteric neurons from adult rat small intestine. Two different *in vitro *models were used. First the ability of the three peptides to enhance neuronal survival was tested on myenteric neurons in primary culture. Next neuroprotective effects of the peptides were tested in an *in vitro *system in which enhanced neuronal cell death was generated by co-culturing myenteric neurons with mast cells. Receptor selectivity was, in both these models, tested by using a GLP1 receptor antagonist (exendin(9-39)amide) and a VIP receptor antagonist (hybrid neurotensin 6-11 and VIP 7-28; hybVIP).

GLP1, GLP2 and VIP were in the present study found to efficiently protect myenteric neurons in two different culture systems. Separate receptors, suggested to be neuronally expressed, were activated. These results strongly point towards a powerful therapeutic promise for these three peptides in the prevention of enteric neuropathy in diseases like diabetes, but also in inflammatory and neurodegenerative diseases.

## Methods

### Animals

Female Sprague-Dawley rats (n = 38, 170-180 g), purchased from Charles River, Sulzfeld, Germany, were used. The rats were allowed to acclimatize to the climate- and light-controlled animal facility for at least 5 days prior execution. Standard rat chow and water were supplied at all times. The experimental design was approved by the animal ethics committee, Lund and Malmö, Sweden. Animals were used in accordance with the European Communities Council Directive (86/609/EEC and 2010/63/EU) and the Swedish Animal Welfare Act (SFS 1988:534).

### Myenteric neuronal cultures

Primary cultures of myenteric neurons were prepared from rat small intestine using a previously described method [21]. In brief, rats were deeply anaesthetized using chloral hydrate (300 mg/kg body weight) and the small intestine was exposed via a midline incision. The longitudinal muscle with attached myenteric ganglia were stripped from approximately 20 cm of the distal small intestine. Stripping was performed aseptically without penetrating the gut thereby avoiding contamination by luminal fecal matter. The tissue was cut into smaller pieces (2 × 2 mm) and washed in Ca^2+^- and Mg^2+^- free Hank's balanced salt solution (HBSS, Gibco, BRL, Life Technologies AB, Stockholm, Sweden). Tissues were placed in HBSS containing collagenase II (1.5 mg/ml, Life Technologies AB) and protease (1.5 mg/ml, Sigma-Aldrich, Stockholm, Sweden) and mechanically separated by trituration 15 times (15×) followed by incubation 25 min at 37°C in a humidified incubator holding 5% CO_2_. Tissue material was vortexed and trypsin (1.25 mg/mL, BioChrom, Berlin, Germany) and EDTA (0.01%, Ethylenediaminetetraacetic acid, Sigma-Aldrich) added and the tissue was again triturated 15× and incubated rotating 20 min at 37°C, 5% CO_2_. The tissue was vortexed and 50% vol/vol fetal calf serum (FCS, Gibco BRL, Life Technologies) was added. The cell suspension was passed through a rough mesh (Ø 1 mm), centrifuged at 7.4 g for 7 min and washed in HBSS, the washing procedure was repeated twice. The cell pellet was diluted to 2.5 mL in Neurobasal A (NBA) culture medium containing 10% FCS, 0.5 mM L-glutamine, 50 U/mL penicillin G sodium and 50 μg/mL streptomycin sulphate (all from Gibco BRL, Life Technologies). Cell cultures were prepared by seeding 50 μl of constantly mixed cell suspension into 8-well chambers (cat no 734-0402, BD Falcon, VWR, Göteborg, Sweden) prefilled with 450 μL NBA and incubated in humidified incubator holding 5% CO_2_. From each rat six 8-wells chambers (each well 69 mm^2^) were prepared; cell cultures were never prepared by pooling cell suspensions from different rats. After 4 days *in vitro *(4 D*IV*), 400 μL of medium was replaced with fresh medium and, depending on experimental set up, mast cells (see below) and/or pharmacological agents were added. The cultures were then grown for 2 days (4 + 2 D*IV*) followed by fixation for 30 minutes in a mixture of 2% formaldehyde and 0.2% picric acid (Stefanini's fixative) in 0.1 M phosphate-buffer, pH 7.2, rinsing twice in Tyrode's solution containing 10% sucrose. In order to enhance antibody penetration, the cultures were frozen in -20°C for at least 1 h before being processed for immunocytochemistry.

Evaluation was by neuronal cell counting. Parallel controls were cultured in NBA (supplemented as described above).

### Isolation of mast cells

Adult female rats (Sprague-Dawley, 250-300 g, n = 16) were killed by cervical dislocation. Mast cells were obtained by peritoneal saline washings using procedures previously described [21]. In brief, peritoneal lavage was performed by injecting 10 mL saline containing heparin (5 U/mL) into the peritoneal cavity followed by abdominal massage for 4 min. The mast cell rich fluid (approximately 7 mL) was removed and centrifuged at 15 g for 2 min at room temperature. Precipitated cells were resuspended in 1 mL NBA.

### Co-culturing myenteric neurons and mast cells

To 4 D*IV *cultures of myenteric neurons 50 μL (= 18 988 ± 1 046 mast cells, mean ± SEM; see section "histochemistry" for details) of mast cell suspension was added. To test if GLP1, GLP2 or VIP inhibited mast cell-induced neuronal cell death the peptides (10^-9 ^- 10^-6 ^M) were added to the neuron-mast cell co-cultures, in the presence or absence of receptor antagonist.

At the end of experimentation, referred to as 4 (neuronal pre-culture period) + 2 (co-culture period) D*IV *the cultures were fixed, rinsed (as described above) and frozen until being processed for immunocytochemistry. Evaluation was by neuronal cell counting after 4 + 2 D*IV*. Parallel controls were cultured in NBA (supplemented as described above) with or without addition of mast cell and without peptides or receptor antagonists.

### Pharmacological agents

To test if the presence of GLP1 (human GLP1(1-36)amide, PolyPeptide Laboratories A/S, Hillerød, Denmark), GLP2 (human GLP2(1-34), PolyPeptide Laboratories A/S) or VIP (rat VIP, Sigma-Aldrich) affected neuronal survival, the peptides were added separately (10^-11^- 10^-6 ^M) to 4 D*IV *cultures or co-cultures.

Possible inhibitory effects of GLP1 receptor antagonist exendin(9-39)amide (10^-7 ^M; Bachem) or VIP receptor antagonist hybVIP (10^-9 ^M; hybrid neurotensin 6-11 and VIP 7-28, Bachem, St. Helens, UK) on GLP1-, GLP2-, or VIP- induced neuroprotection were tested by addition of the receptor antagonist simultaneously with the peptide studied. All substances were dissolved in sterile water.

### Histochemistry

In order to estimate the number of mast cells seeded in each well, 50 μL of the mast cell suspension was placed on a glass slide. Two such slides were performed from each rat. The slides were dried (30 min in 37°C), fixed and rinsed as described above. To detect mast cells the slides were stained 10 min with 0.1% toluidine blue (CI 52 040). Mast cells become deep purple due to metachromasia. The number of mast cells per slide was estimated by computerized morphometry [21]. The total number of mast cells seeded (in 50 μL mast cell suspension) was found to be 18 988 ± 1 046 mast cells (mean ± SEM; n = 4).

### Immunocytochemistry

As general neuronal markers antibodies against HuC/HuD (a mouse monoclonal anti-human neuronal protein; code no A-21271; Molecular Probes, Eugene, OR, USA; dilution 1:400; [22]) or human protein gene product 9.5 (raised in rabbit, code no PGP9.5; Ultraclone, Isle of Wight, UK; dilution 1:1600; [21]) were used. In terms of number of nerve cell bodies visualized *in vitro *the HuC/HuD and the PGP 9.5 antibodies gave identical results, as previously described [23]. Mast cells were detected by antibodies raised in rabbits against histamine coupled to human serum albumin using carbodiimide (code no 8432, Euro-Diagnostica AB, Malmö, Sweden; dilution 1:300; [24]).

Immunolabeling was done by incubation of the slides with primary antibodies in a moist chamber over night at 4°C. For visualization, the slides were exposed to fluorescein isothiocyanate (FITC)- or Texas Red- conjugated goat anti-rabbit IgG antiserum (Jackson Immunoresearch Laboratories, West Grove, PA, USA; dilution 1:100) or FITC conjugated goat anti-mouse IgG antiserum (Jackson Immunoresearch Laboratories; dilution 1:100) for 1 h, mounted in phosphate buffer:glycerol 1:1 and analyzed using a fluorescence microscope with appropriate filter settings. Since synthetic antigens for testing the specificities of the HuC/HuD and PGP9.5 are not commercially available, omission of primary antibodies was used as control. In order to assess the specificity of the histamine antiserum 10 μM histamine was incubated with the antibodies (dilution 1:300) over night before being exposed to the slides. Controls did not exhibit any immunostaining.

### Cell counting and statistical analysis

Neuronal survival was calculated by counting the numbers of surviving neurons in the entire culture chamber after exposure to mast cells and/or pharmacological agents and expressed in percentage of the control run in parallel.

Statistical differences were determined using one-way analysis of variance test (ANOVA) followed by Dunnett's multiple comparison test (all comparisons against control column). Statistical analysis was performed using GraphPad Prism 5 (GraphPad Software Inc., San Diego, CA, USA)

## Results

### Myenteric neurons in culture and in co-culture with mast cells

**Figure 1:**
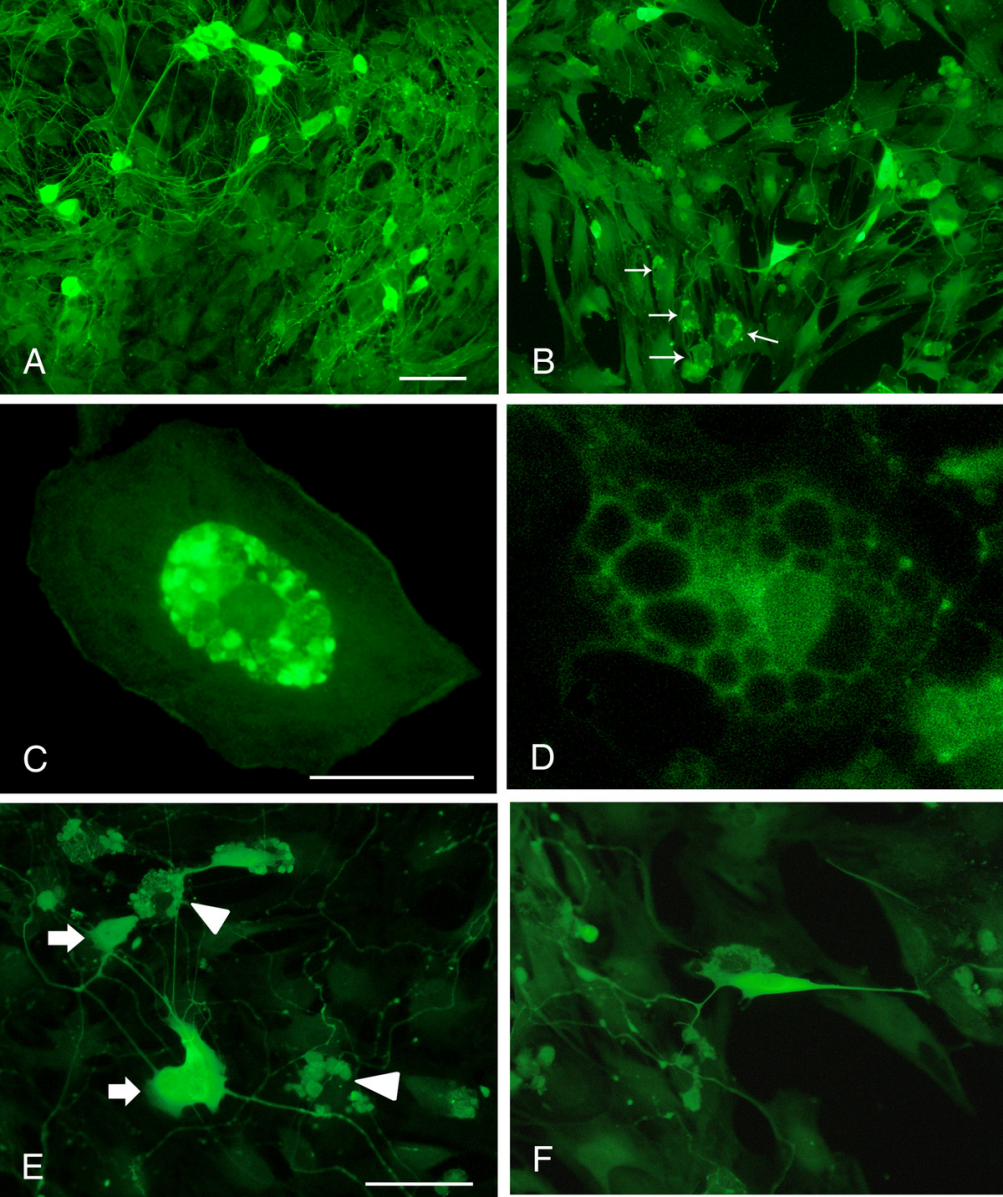
**Immunocytochemical staining of myenteric neurons cultured in absence (A) or presence (B-F) of peritoneal mast cells**. A. Neurons grown for 6 days and stained with PGP9.5. The cultured neurons survive well; they group into ganglion-like structures and grow a prominent arborizing network of nerve terminals. B. Neurons co-cultured with mast cells (arrows) stained with antibodies against PGP9.5 and histamine, respectively. In the neuronal cultures to which mast cells have been added the number of neurons markedly decreases and the terminal network is disintegrated. C and D. show mast cells grown in co-culture and stained with histamine antibodies. The mast cells are well settled within the cultures and they contain a high number of cytoplasmic granules. C. illustrates a cultured mast cell with numerous granules in the central, perinuclear, portion of the cell. D. shows a mast cell exhibiting piece meal degranulation. E and F. illustrate the morphological relationship of cultured neurons and mast cells. E. shows mast cells (arrowheads) in close contact with nerve terminals from nearby myenteric neurons (arrows). F. a mast cell and a nerve cell body in close proximity. In order to perceive the morphological arrangement of mast cells and neurons both PGP9.5- and histamine- immunoreactive cells are visualised by FITC conjugated antibodies. Neurons and mast cells are distinguished by their morphological characteristics. Bar in A 100 μm represents also B, bar in C 40 μm represents also D, bar in E 50 μm represents also F.

Neurons cultured for 4 + 2 days stained well with both HuC/HuD and PGP9.5 and were uniformly dispersed throughout the culture wells. Neurons with rich networks of arborizing fibers were displayed after staining with PGP 9.5 antibodies (Figure 1A), while HuC/HuD antibodies revealed nerve cell bodies only. The number of neurons was 4.9 ± 0.4 neurons/mm^2 ^(n = 24), as established on parallel controls.

Addition of mast cells to pre-cultured (4 D*IV*) myenteric neurons resulted in a disintegrated nerve fibre network and a markedly enhanced loss of nerve cell bodies (Figure 1B). After 2 days in co-culture (4 + 2 D*IV*) the numbers of myenteric neurons were reduced to 2.4 ± 0.2 neurons/mm^2 ^(n = 24), *p *< 0.01 as compared to neurons grown in parallel without the addition of mast cells.

Within the co-cultures mast cells dispersed uniformly, survived well and adhered to the bottom of the culture chambers. When stained with histamine antibodies or toluidine blue the mast cells were found to contain numerous granules within the cytoplasm (Figure 1C). Cultured mast cells often showed signs of degranulation and some exhibited piece meal degranulation (Figure 1D). Mast cells and neurons were occasionally seen in close proximity (Figures 1E and 1F).

### Survival of neurons after GLP1, GLP2 or VIP treatment

Myenteric neurons pre-cultured for 4 days, followed by exposure to GLP1, GLP2 or VIP in different concentrations (10^-11^-10^-6 ^M) for 2 days (4 + 2 D*IV*), showed a markedly increased neuronal survival, compared to untreated controls run in parallel. The increase was concentration-dependent for all three peptides. EC_50 _(M) values were 3 × 10^-11 ^for GLP1, 3.5 x10^-10 ^for GLP2 and 4 × 10^-11 ^for VIP. Emax (%; maximal neuronal survival as a percentage of control) values were 178.5 ± 14.0 for GLP1, 150.8 ± 6.0 for GLP2 and 150.4 ± 8.4 for VIP.

**Figure 2:**
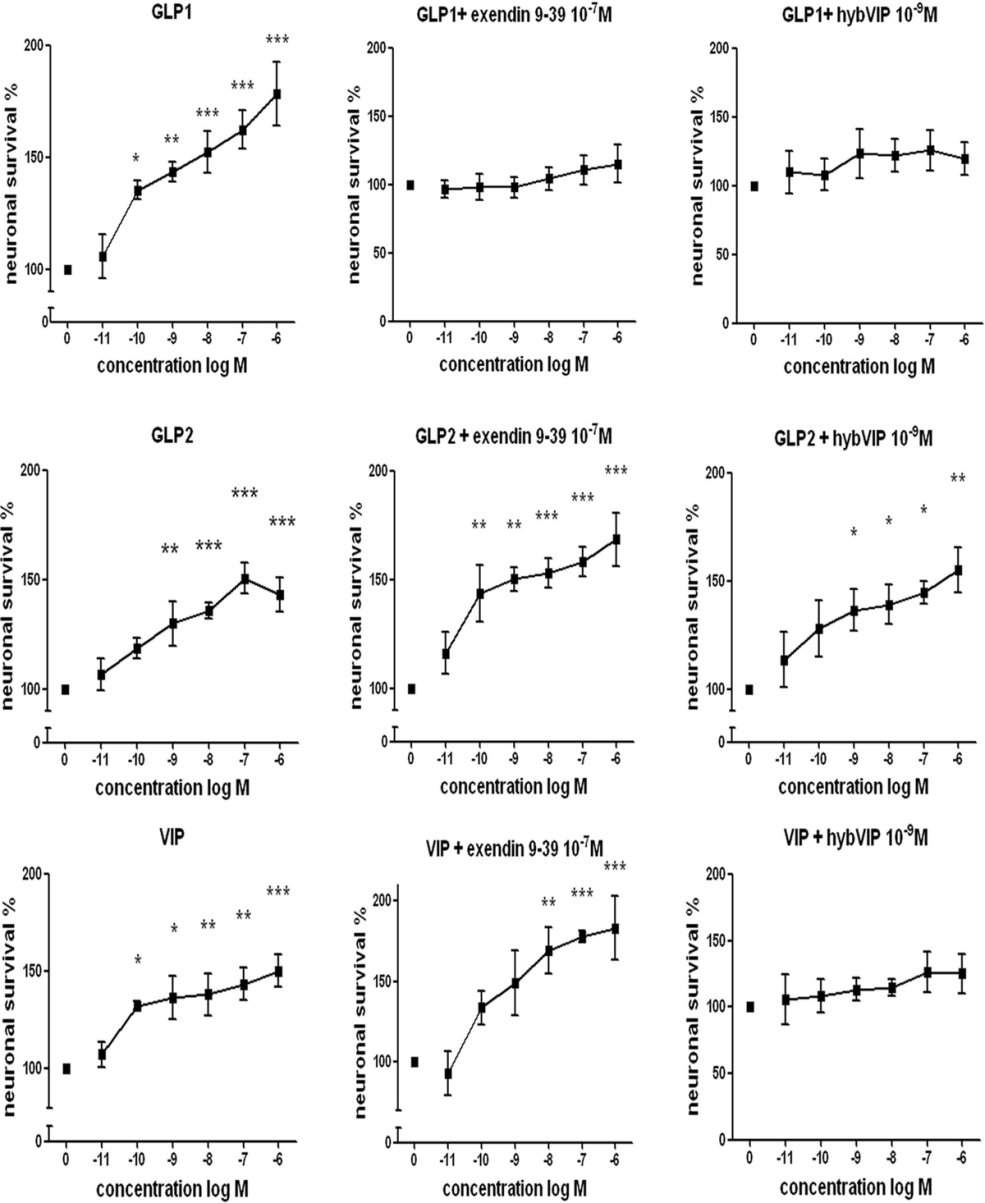
**Survival of myenteric neurons pre-cultured for 4 days followed by 2 days of culture in various concentrations (10^-11^- 10^-6 ^M) of GLP1, GLP2 or VIP in the absence or presence of GLP1 receptor antagonist (exendin(9-39)amide, 10^-7 ^M) or VIP receptor antagonist (hybVIP, 10^-9 ^M)**. Presence of GLP1, GLP2 and VIP significantly and concentration-dependently increase neuronal survival. Simultaneous addition of exendin(9-39)amide attenuates GLP1-induced neuroprotection while that of GLP2 and VIP are unaffected. Simultaneous addition of hybVIP attenuates GLP1- and VIP-induced neuroprotection while that of GLP2 is unaffected. Means ± SEM, n = 6-21; * *p *< 0.1; ** *p *< 0.05; *** *p *< 0.01 as compared to controls run in parallel without addition of peptides or antagonists.

Peptide receptor selectivity was tested in experiments using receptor antagonists for either GLP1 (10^-7 ^M, exendin(9-39)amide) or VIP (10^-9 ^M, hybVIP) receptors added simultaneously with the different peptides. In these experiments both the GLP1- and the VIP- receptor antagonists inhibited GLP1-induced increases in neuronal survival. GLP2-induced survival was unaffected by the addition of any of the two antagonists. VIP-induced survival was unaffected by the addition of exendin(9-39)amide but markedly attenuated by the addition of hybVIP. The results are summarized in Figure 2.

### Survival of neurons co-cultured with mast cells in the presence of GLP1, GLP2 or VIP

**Figure 3:**
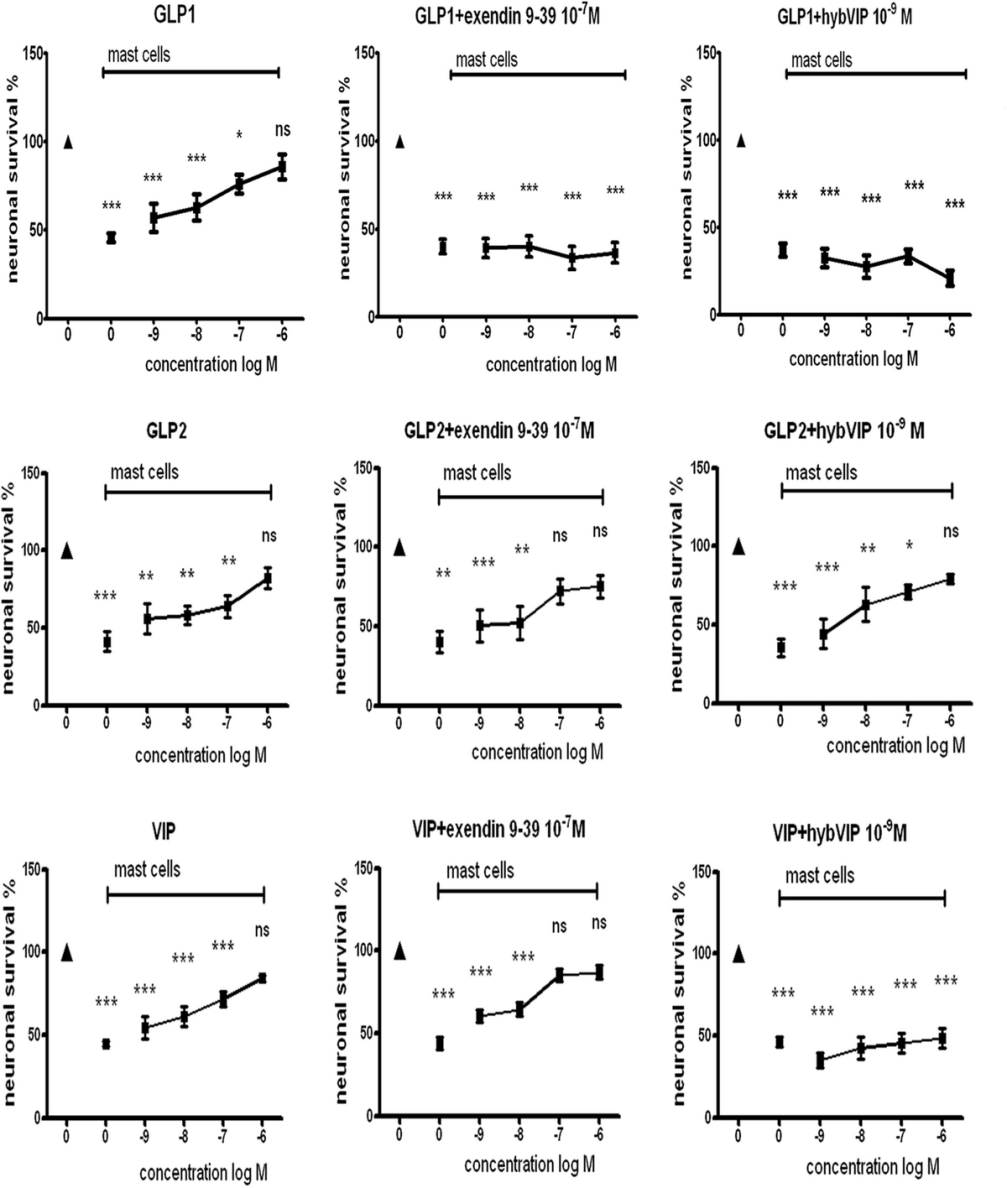
**Survival of neurons pre-cultured for 4 days followed by 2 days of culture in the absence (▲) or presence of mast cells (■, indicated by horizontal bar in each graph)**. During the co-culture period the effects of GLP1, GLP2 and VIP (10^-9^- 10^-6 ^M), in the absence or presence of GLP1 receptor antagonist (exendin(9-39)amide, 10^-7 ^M) or VIP receptor antagonist (hybVIP, 10^-7 ^M), on neuronal survival were determined by neuronal cell counting. With increasing concentrations all three peptides were able to reverse mast cell-induced neuronal cell death (denoted ns; no significant difference). Simultaneous presence of exendin(9-39)amide (10^-7 ^M) counteracted the effects of GLP1 while GLP2- or VIP-induced neuroprotection was unaffected. Addition of hybVIP (10^-9 ^M) to the co-cultures hampered GLP1- and VIP-, but not GLP2-, induced neuroprotection. Means ± SEM, n = 5-12; ns, no significant difference; * *p *< 0.1; ** *p *< 0.05; *** *p *< 0.01 as compared to controls run in parallel (▲) without addition of mast cells, peptides or antagonists.

Presence of GLP1, GLP2 or VIP (10^-9^-10^-6 ^M) within the co-cultures of myenteric neurons and mast cells significantly and concentration-dependently enhanced neuronal survival. At the highest concentration (10^-6 ^M) all three peptides were able to completely reverse mast cell-induced neuronal cell death, as compared to neurons cultured in parallel in the absence of mast cells. Simultaneous presence of exendin(9-39)amide (10^-7 ^M) counteracted the effects of GLP1 while GLP2- or VIP-induced enhanced neuronal survival was unaffected. Addition of hybVIP (10^-9 ^M) to the co-cultures hampered both GLP1- and VIP-induced neuroprotection. Results are summarized in Figure 3.

## Discussion

### GLP1, GLP2 and VIP promote neuronal survival

Our studies show that GLP1, GLP2 and VIP all were able to enhance survival of myenteric neurons in culture. Further all three peptides counteracted mast cell-induced neuronal cell death. Neuroprotection induced by GLP1 was reversed by either the addition of the GLP1 receptor antagonist, or the VIP receptor antagonist. GLP2-induced neuroprotection was unaffected by both the receptor antagonists, and VIP-induced neuroprotection was attenuated only by the VIP receptor antagonist. Taken together these results indicate that GLP1, GLP2 and VIP receptors are operating to promote neuronal survival in the ENS. GLP1 receptors are suggested to be localized on VIP-expressing myenteric neurons and to release VIP on activation, or act on a parallel pathway which converges with VIP-signaling to a common final mechanism. This is supported by the fact that GLP1-induced neuroprotective responses were blocked by the addition of the GLP1 receptor blocker exendin(9-39)amide, as well as by VIP receptor antagonist. VIP and GLP2 receptors are suggested to evoke direct protective effects on myenteric neurons upon activation.

The peptide concentrations studied verified the EC50 values to be clearly within the *in vivo *plasma concentration range in both man and rat as shown for GLP1 [25,26], GLP2 [25,27] and VIP [28,29].

### VIP receptors

The finding that VIP promotes neuronal survival of myenteric neurons *in vitro *is well in line with several previous observations [16,17,19,20]. Also the finding that the presence of VIP rescues myenteric neurons from mast cell-induced death is supported by previous findings [21]. VIP-mediated neuroprotective effects are in addition corroborated by several other studies performed in neurons belonging to the central nervous system [reviewed in [18]]. The receptor(s) by which VIP mediates neuroprotection is, however, enigmatic. Cloning of receptors for the VIP superfamily of peptides has revealed three distinct receptors activated by VIP; VPAC_1_, VPAC_2 _and PAC_1 _receptors [30]. All three are in addition high affinity receptors for pituitary adenylate cyclase-activating peptide (PACAP). Therefore the report that, in contrast to VIP, presence of PACAP does not elicit any neuroprotective effect on cultured rat myenteric neurons [19] led to the suggestion of "VIP specific" receptors. In the present study we have used a commercially available competitive antagonist of VIP-binding. The antagonist, a chimeric peptide consisting of neurotensin (6-11) and VIP (7-28), was found to antagonize the "VIP specific" receptor mediated neuroprotective effect on cultured rat myenteric neurons. Thus, this antagonist is suggested to have a broad spectrum resembling (N-stearyl, norleucine17)VIPhybrid which recognizes both VPAC_1 _and VPAC_2 _receptors as well as PAC_1 _receptors [31].

### GLP1 receptors

GLP1 receptor stimulation is widely used clinically to improve glucose homeostasis in patients with type 2 diabetes [6,32] but has also been recognized to mediate neuroprotection and neurotrophic effects. GLP1 was first described to protect central neurons against excitotoxicity [33,34] but later also to have neuroprotective properties in neurodegenerative diseases like Alzheimer's and Parkinson's diseases [9]. Protective effects of GLP1 on peripheral neurons have so far only been described in sensory neurons [10,11].

A single GLP1 receptor, similar in rodents and man, has been cloned [35]. It is a 7-transmembrane G-protein coupled receptor with structural similarities to the GLP2 receptor. GLP1 receptors are widely expressed throughout the body in e.g. digestive tract, kidney and in peripheral as well as central neurons [35]. Myenteric neurons in both small and large intestine of mice express GLP1 receptors [36]. In the present study we suggest, based on the ability of both hybVIP and exendin(9-39)amide to antagonize the GLP1-induced neuroprotective effects, that GLP1 binds to GLP1 receptors localized on VIP-expressing myenteric neurons, thereby causing release of VIP. The thus released VIP is responsible for GLP1-induced neuroprotection of cultured myenteric neurons.

The finding that GLP1, by way of VIP release, executes neuroprotection on myenteric neurons highlights the possibility of additional beneficial effects activated by the use of GLP1 receptor stimulation to achieve better metabolic control in type 2 diabetes clinically. Such treatment would provide neuroprotection of the ENS which may be speculated to attenuate diabetic neuropathy in the GI tract. In this context it is of interest also to note the fact that enteric myenteric neurons issue intestinofugal projections to pancreatic ganglia [37] and that VIP stimulates insulin release from pancreatic islets [38]. GLP1 stimulation of VIP-expressing enteric neurons may thus be yet another mechanism by which GLP1 analogue-based therapies execute beneficial effects on insulin release and glucose homeostasis.

### GLP2 receptors

Within the GI tract GLP2 has significant intestinotrophic effects involving diverse cellular organisations despite the finding that GLP2 receptors are mainly localized on enteric neurons, endocrine cells and subepithelial myofibroblasts [39]. The restricted GLP2 receptor distribution has led to the proposal that the multitude of GLP2-induced effects is mediated by indirect mediators and diverse signaling pathways [40]. One such mediator is VIP. In mouse, GLP2-induced gastric relaxation is executed by prejunctional neuronal VIP release [41]. GLP2 has further been found to attenuate inflammation-induced intestinal damage and levels of proinflammatory cytokines in animal models of intestinal inflammation via release of VIP [14,15]. GLP2 further prevents loss of submucous neurons in inflamed bowel and increases the proportion of VIP-expressing neurons within submucous ganglia in both inflamed and non-inflamed bowel [15]. The neuroprotective effect of GLP2 demonstrated *in vivo *on submucous neurons is well in agreement with our present *in vitro *findings indicating significantly improved neuronal survival and a rescue from mast cell-induced neuronal cell death of cultured myenteric neurons when exposed to GLP2. The data presented here suggests that GLP2-induced neuroprotection of myenteric neurons is mediated by direct stimulation of neuronal GLP2 receptors and not executed by VIP release or by activation of GLP1 receptors. This since the GLP2-induced increase in neuronal survival was unaffected by the presence of hybVIP and exendin(9-39)amide. However, we cannot definitely exclude indirect mediators derived from e.g. enteric glia. Such cells are unavoidably present within the primary cultures of myenteric neurons as well as within the co-cultures consisting of mast cells and neurons. A similar dilemma concerning direct *vs *indirect GLP2 effects is faced by the *in vivo *studies on experimental colitis [15]. In these studies the GLP2 neuroprotective effect on submucous neurons was suggested to be either direct or due to the general anti-inflammatory effects caused by systemic GLP2 treatment. In addition to enteric neurons GLP2-mediated neuroprotective effects have also been demonstrated in central, i.e. hippocampal, neurons [42,43] signifying that GLP2 receptor stimulation may promote survival in different types of neurons.

### GLP1, GLP2 and VIP rescue neurons from mast cell-induced cell death

Although mast cells have been ascribed a role in the metabolic syndrome and type 2 diabetes pathophysiology [44] the main reason for testing neuronal survival in co-cultures of myenteric neurons and mast cells was to test VIP, GLP1 and GLP2 effects in an *in vitro *setting in which neuronal cell death was actively enhanced. Mast cells have been shown to induce death of myenteric neurons *in vitro *by way of degranulation and also by release of mediators not stored within granula. Prostaglandin D_2 _and interleukin-6, known mast cells mediators, induce death of myenteric neurons *in vitro *as do proteinase-activated receptor_2 _activation [21]. The mechanisms by which GLP1, GLP2 and VIP rescue myenteric neurons from mast cell-induced cell death needs further examination but is probably executed by direct activation of neuronal receptors, as in neuronal cultures without the addition of mast cells. The effects of the peptides are mediated via activation of separate receptors in both experimental models used in the present study. It may be speculated that GLP1, GLP2 and VIP, in the co-cultures, have direct e.g. stabilizing effects on mast cells but present data does not substantiate such speculations. Whether mast cells express GLP1 or GLP2 receptors is at present unknown. Human mast cells in culture express VIP receptors of VPAC_2 _type which, upon activation, cause mast cell degranulation [45]. Given that this applies also to cultured rat mast cells then VIP exposure would be suspected to cause an even more severe neuronal cell loss within the neuron-mast cell co-cultures. In this context it is noteworthy that brain mast cells, upon VIP activation, are suggested to participate in central neuronal protection by adopting a nondegranulating phenotype [46]. Thus the detailed mechanisms behind the VIP-induced neuroprotection, as well as that induced by the GLPs, are still unresolved.

## Conclusions

The neuroprotective effects of the GLPs and VIP on myenteric neurons are powerful and worthy of further exploration in view of the large number of patients facing enteric neuropathy. Neuroprotection of the ENS is in the present work recognized as yet another positive effect resulting from incretin-based therapies.

## Abbreviations

ANOVA: analysis of variance; D*IV*: days *in vitro*; EDTA: ethylenediaminetetraacetic acid; ENS: enteric nervous system; FCS: fetal calf serum; FITC: fluorescein isothiocyanate; GI: gastrointestinal; GLP1: glucagon like peptide 1; GLP2: glucagon like peptide 2; HBSS: Hank's balanced salt solution; HuC/HuD: human neuronal proteins HuC and HuD; hybVIP: hybrid neurotensin 6-11 and vasoactive intestinal peptide 7-28; IBD: inflammatory bowel disease; NBA: culture medium neurobasal A; NO: nitric oxide; NOS: nitric oxide synthase; PACAP: pituitary adenylate cyclase-activating peptide; PAC_1_: VPAC_1 _and VPAC_2 _receptors: receptors for vasoactive intestinal peptide and pituitary adenylate cyclase activating peptide; PGP9.5: protein gene product 9.5; TUNEL: terminal deoxynucleotidyl transferase dUTP nick end labelling; VIP: vasoactive intestinal peptide.

## Competing interests

The authors declare that they have no competing interests.

## Authors' contributions

UV, ES and EE performed the experimentation. EE and UV analyzed data and wrote the manuscript. PH provided GLP and participated in the writing of the manuscript. EE conceived and designed the study. All authors read and approved the final manuscript.

## Pre-publication history

The pre-publication history for this paper can be accessed here:

http://www.biomedcentral.com/1471-230X/12/30/prepub
